# Secondary primary malignancies during the lenalidomide–dexamethasone regimen in relapsed/refractory multiple myeloma patients

**DOI:** 10.1002/cam4.799

**Published:** 2016-11-18

**Authors:** Rouslan Kotchetkov, Esther Masih‐Khan, Chia‐Min Chu, Eshetu G. Atenafu, Christine Chen, Vishal Kukreti, Suzanne Trudel, Rodger Tiedemann, Donna E. Reece

**Affiliations:** ^1^ Medical Oncology and HematologyPrincess Margaret Cancer CentreTorontoONCanada; ^2^ Department of BiostatisticsPrincess Margaret Cancer CentreTorontoONCanada; ^3^ McLaughlin Centre for Molecular MedicinePrincess Margaret Cancer CentreTorontoONCanada

**Keywords:** AML, lenalidomide, MDS, relapsed/refractory multiple myeloma, secondary malignancies

## Abstract

Lenalidomide in combination with dexamethasone (Len‐dex) represents a highly effective treatment in relapsed/refractory multiple myeloma (RRMM) patients. However, an increased risk of secondary primary malignancies (SPMs), including myelodysplastic syndrome (MDS) and acute myelogenous leukemia (AML) has been described in patients receiving lenalidomide. In order to assess the incidence and features of this complication, we reviewed 195 patients with RRMM treated with Len‐dex at our institution. The median follow‐up time from diagnosis of MM was 73 months (10–234 months) and from initiation of Len‐dex was 19 months (1–104 months). The median duration of Len‐dex for all patients was 7.8 months (range 1–90 months). The incidence rate (IR) for all SPMs from start of Len‐dex was 2.37 per 100 patient‐years, which reflected an IR of 1.29 for MDS/AML and 1.08 for nonhematologic malignancies (NHM). MDS was the most common SPM noted. The cumulative IR of SPM at 5 years was 1.54% from the time of MM diagnosis and 5.24% from starting Len‐dex. Multivariable cumulative incidence of SPM analysis identified older age (*P* = 0.005) and prior number of regimens (*P* = 0.026) as adverse risk factors. We found more concomitant G‐CSF use (*P* = 0.029) in patients with MDS/AML, however, causal association is not clear. The progression‐free survival after Len‐dex was the longest for patients in MDS/AML group, and the 5‐year overall survival did not differ among groups. Although the rate of SPM was relatively low with Len‐dex, concomitant G‐CSF should be used judiciously and patients receiving this regimen should be observed for the development of this complication.

## Introduction

The introduction of new agents, primarily immunomodulatory agents and proteasome inhibitors, has changed the course of multiple myeloma (MM) from a fatal disease with short life expectancy to a chronic cancer characterized by sequential remissions and relapses that, in turn, require multiple lines of treatment. Therefore, an increasing number of MM patients are on protracted anti‐myeloma therapy and are living longer. Concomitant with this observation, there is emerging data describing an increased risk of developing secondary primary malignancies (SPMs) in MM survivors 1, 2.

The combination of lenalidomide and dexamethasone (Len‐dex) is well‐tolerated and produces significant survival benefits in heavily pretreated, relapsed and/or refractory multiple myeloma (RRMM) patients 3. Two large phase 3 trials (MM‐009 and MM‐010) showed that Len‐dex prolonged both progression‐free and overall survival (OS) compared with placebo plus dexamethasone (OS: 38 vs. 31.6 months, *P* = 0.045) after a median follow‐up of 48 months 3, 4, 5, 6, 7. Currently the Len‐dex combination is a standard treatment option for RRMM. However, concerns have been raised about the potential for an increase in SPMs in myeloma patients exposed to lenalidomide, particularly in the maintenance setting 8.

Recently, an increased incidence of invasive SPMs has been observed with lenalidomide (7.8%) compared with controls (2.9%) in patients with newly diagnosed MM receiving lenalidomide in combination with melphalan 9 or as long‐term maintenance therapy after high‐dose melphalan with autologous stem cell transplant (ASCT) 10, 11. In the setting of RRMM, an analysis of pooled data from the pivotal phase 3 trials with 703 patients reported that the overall incidence rate (IR) (events per 100 patient‐years) of SPMs was 3.98 with Len‐dex versus 1.38 with placebo/dexamethasone. IRs of nonmelanoma skin cancers were 2.40 and 0.91, respectively; IRs of invasive SPMs were 1.71 and 0.91, respectively 12.

Between 2006 and 2009, almost 200 relapsed/refractory patients seen at the Princess Margaret Cancer Center (PMH) received therapy with Len‐dex, and we have conducted a retrospective review of our patients to identify the incidence and characteristics of SPM, including acute myelogenous leukemia (AML)/myelodysplastic syndrome (MDS), in this population.

## Patients and Methods

### Patients

We used the clinical Multiple Myeloma Database at PMH to identify patients who received treatment with Len‐dex for RRMM with at least one prior regimen. The eligible patients had no prior exposure to lenalidomide as primary or maintenance therapy. The MDS and AML diagnosis for patients was made with a bone marrow aspirate and cytogenetics/fluorescent in situ hybridization (FISH). Retrospective chart review of these patients was conducted to determine the incidence and features of SPMs that developed during this therapy. Approval for the review of these records was obtained from the PMH Institutional Review Board and was in accordance with the Declaration of Helsinki.

### Statistical analyses

SPMs were defined using the Medical Dictionary for Regulatory Activities (MedDRA) terms found under the System Organ Class “Neoplasms.” IRs were defined as events per 100 patient‐years, and their confidence intervals (CIs) were calculated. Patient‐year was defined as the time in years from the first dose of Len‐dex to SPM onset for patients with an SPM, and the time from the first dose to the last dose for patients without an SPM. Overall IRs included invasive SPMs, defined as hematologic or solid tumor malignancies. Responses to treatment and disease progression were assessed with the use of modified European Group for Blood and Marrow Transplantation criteria. Progression‐free survival (PFS) was defined in months as the time from the start of therapy to disease progression or death, while OS was defined from the date of MM diagnosis to the date of death from any cause 13.

Categorical variables such as the incidence of secondary malignancy, gender, history of radiotherapy, prior therapy, type of SPM, and best response to Len‐dex were summarized with counts and percentages. Continuous variables such as age at diagnosis and at start of treatment, as well as time to events, were summarized with means, standard deviation (SD), medians and/or ranges as appropriate. Fisher's exact test was used to compare categorical covariates and log‐rank test used for comparing continuous variables of interest among diagnosis types. The main outcome variable of interest included time to incidence of SPMs. Other secondary outcome measures included time to progression on Len‐dex and time to death.

OS and PFS rates were calculated using the Kaplan–Meier product‐limit method. Fine and Gray's method for competing risk was used for cumulative incidence of SPM considering death as a competing risk. All *P*‐values were two‐sided and for the statistical analyses, *P* < 0.05 was considered to indicate a significantly different result. Data analysis was performed using (SAS) Version 9.4 (SAS Institute, Inc., Cary, NC) and the open source statistical software R version 3.0.0 (R Core Team (2013), R Foundation for Statistical Computing, Vienna, Austria (URL http://www.R-project.org/).

## Results

### Patient characteristics

All 195 RRMM patients who received at least one cycle of Len‐dex between the years of 2006–2009 at PMH were included in the analysis. The median duration of lenalidomide treatment was 7.8 months (range 1–90 months). The median patient age at start of lenalidomide was 61 years (range 31–80 years), while 57% were male. The majority of patients had received two or more prior MM treatments (median 2, range 1–7) that included corticosteroids (88%), thalidomide (64%), oral cyclophosphamide (69%), and bortezomib (42%) (Table 1).

**Table 1:** Summary of patient characteristics for AML/MDS, NHM, and non‐SPM groups

Features	All patients (*N* = 195)	AML/MDS (*N* = 6)	Nonhematological cancers (*N* = 5)	AML/MDS/other cancers negative (*N* = 184)
Median age (range) at start of lenalidomide	61 (31–80)	66.5 (53–76)	66 (62–68)	61 (31–80)
Median age (range) at MM diagnosis	57 (31–77)	63.1 (52.1–69.4)	60.8 (57.4–67.3)	57.1 (30.8–77.4)
Male, *n* (%)	112 (57)	3 (50)	2 (40)	107 (58)
Median baseline ANC (range), × 10^9^/L	2.8 (0.9–61.4)	2.6 (1.8–3.5)	1.6 (0.9–3.5)	2.8 (0.9–61.4)
Median baseline platelet count (range), × 10^9^/L	155 (5–420)	208.5 (43–277)	166 (100–208)	153 (5–420)
IgG, *n* (%)	112 (57.4)	4 (67)	3 (60)	105 (57)
IgA, *n* (%)	37 (19)	2 (33)	1 (20)	34 (19)
FLC, *n* (%)	41 (21)	0 (0)	0 (0)	41 (22)
Nonsecretory, *n* (%)	5 (2.6)	0 (0)	1 (20)	4 (2.2)
17p deletion/tested patients, *n* (%)	4/31 (13)	–	–	4/29 (14)
Median creatinine at start of Len (range), mmol/L	87 (39–515)	98.5 (56–117)	69 (52–115)	87 (39–515)
Median LDH (range) at start of Len, U/L	208 (51–1923)	217.5 (51–492)	155 (84–266)	208 (63–1923)
Median Len treatment duration in months (range)	7.8 (0.7–90.3)	23.2 (6.6–56.5)	30.5 (12.1–81.9)	6.9 (0.7–90.3)
Median number of Len cycles	8	25	32	7
Best Response on Len therapy ≥ VGPR, *n* (%)	47 (24.11)	3 (50)	3 (60)	41 (22)
Median number of prior regimen (range)	2 (1–7)	1.5 (1–3)	3 (2–3)	3 (1–7)
Prior ASCT – none, *n* (%)	40 (20.5)	2 (33)	1 (20)	37 (20)
Prior ASCT – one, *n* (%)	124 (63.6)	3 (50)	3 (60)	118 (64)
Prior ASCT – two, *n* (%)	30 (15.4)	1 (17)	1 (20)	28 (15)
Prior oral alkylator, *n* (%)	151 (77.4)	3 (50)	4 (80)	144 (78)
Median prior oral alkylator exposure in days (range)	133 (0–1270)	210.5 (0–628)	130 (0–400)	134 (0–1270)
Prior thalidomide, *n* (%)	124 (63.6)	2 (33)	4 (80)	118 (64)
Prior bortezomib, *n* (%)	82 (42.1)	1 (17)	1 (20)	80 (43)
G‐CSF use during Len treatment, *n* (%)	112 (57.4)	5 (83)	5 (100)	102 (55)
Prior radiotherapy *n* (%)	50 (25.6)	2 (33)	2 (40)	46 (25)

### Incidence of SPMs

Eleven patients developed SPM, for a raw incidence of 5.64% (Tables 1 and 2). Six patients (3.07%) developed AML/MDS, including AML in one patient (0.51%) and MDS in five patients (2.56%). Five patients (2.56%) were diagnosed with a nonhematological malignancy (NHM). NHM group included adenocarcinoma of the rectum, cholangiocarcinoma, urethral cancer, and two patients with squamous cell carcinoma (one of the head and neck and the second of the tonsil). Median follow‐up was 6 years (range 1–20 years) from MM diagnosis and 19 months (range 1–104 months) from initiation of the Len‐dex regimen. Time duration from last ASCT to initiation of Len‐dex regimen was a median of 42 months (range 12–58 months).

**Table 2:** Characteristics of patients with secondary primary malignancies

SPM	Gender	Age Dx of SPM	Time from Dx to SPM (years)	Time from last ASCT to start of Len‐dex (months)	Duration from start Len to SPM Dx (months)	Duration from SPM Dx to death (months)	No. of prior ASCT	Radiation prior SPM (site)	Smoking history	Oral cyclo exposure prior SPM (months)
MDS/RAEB‐2	Male	72	5	N/A	19	10	0	Yes (Spine)	N/A	13.9
MDS/SCC	Female	79	10	N/A	34	1	0	Yes (Spine)	N/A	20.2
MDS/PCL	Male	56	4	12	23	7	1	No	Yes	0
MDS	Female	73	8	57	19	13	1	Yes (Spine)	No	20.7
MDS	Male	63	2	23	2	5	2	No	Yes	0
AML	Female	66	10	41	57	7	2	Yes (Spine)	Yes	0
Adenocarcinoma of rectum	Male	65	6	29	31	4	1	Yes (colon)	No	0
Cholangiocarcinoma	Female	66	5	43	15	2	1	Yes (lumbar spine)	Yes	2.4
SCC of Tonsil	Female	70	7	43	38	4	1	N/A	Yes	12.2
Urethral cancer	Female	72	15	58	70	11 (Alive)	2	Yes (extra‐medullary disease)	N/A	4.3
SCC of head and neck	Male	75	8	N/A	79	1 (Alive)	0	No	No	13.2

SPM, secondary primary malignancies; N/A, not available; Dx, diagnosis; PCL, plasma cell leukemia; MDS, myelodysplastic syndrome; SCC, squamous cell carcinoma; ASCT, autologous stem cell transplant; RAEB, refractory anemia with excess of blasts.

The IR for all SPMs per 100 patient‐years was 0.86 from diagnosis and 2.37 from start of Len‐dex treatment. The IR for MDS/AML from start of treatment was 1.29, while the IR for NHM was 1.08.

### All SPMs

The baseline characteristics of all patients, patients who developed any SPM–MDS/AML or NHM versus patients who did not develop SPM are presented in Table 1. Patients who developed a SPM were older at time of diagnosis with a median age of 61 years (range 52–69 years) versus a median of 57 years (range 31–80 years) for patients who did not develop SPM (*P* = 0.043). They were also older at the start of lenalidomide therapy with a median age of 66 (range 53–76 years) versus 61 years (31–80 years, *P* = 0.0329). The groups also marginally differed with respect to the isotype of MM, specifically, the MDS/AML cohort included IgG 67%, IgA 33%, and free light chain (FLC) – only 0% compared to 57%, 19%, and 22% in the non‐SPM group, respectively (*P* = 0.100). The baseline median absolute neutrophil count (ANC) and platelet count as well as baseline creatinine and LDH levels did not differ between the two groups.

MDS/AML and NHM patients combined had received more cycles and had a longer median lenalidomide exposure of 23.6 months (range 7–82 months) versus 6.9 months (range 1–90 months) in non‐SPM patients (*P* = 0.002). Also MDS/AML and NHM patients manifested a better response rate to lenalidomide therapy, with 55% versus 22% (*P* = 0.025) achieving very good partial response (VGPR) or better.

**Figure 1:**
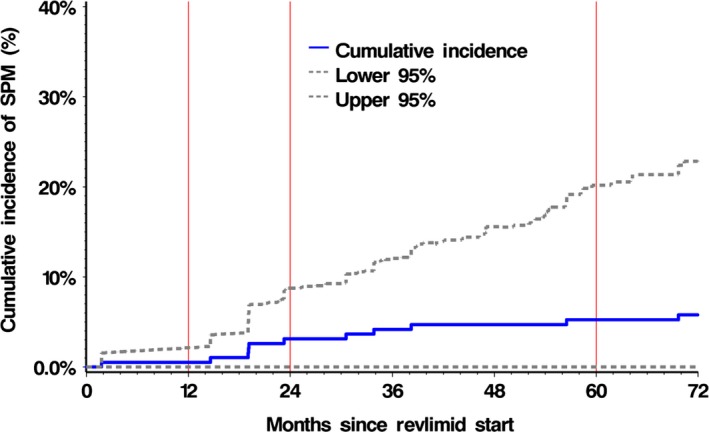
Cumulative incidence of SPM from start of lenalidomide. Two‐year cumulative incidence rate was 3.12% (95% CI, 0–8.5%).

Further analysis of factors related to the cumulative incidence rate (CIR) was performed using the Fine and Gray method for comparing the cumulative incidence of SPMs considering death as a competing risk (Fig. 1). Univariate analyses identified the following factors to have prognostic significance: age at diagnosis (*P* = 0.003), age at start of lenalidomide (*P* = 0.007), absolute neutrophil count (ANC) at start of Len‐dex therapy (*P *= 0.052), G‐CSF use during Len‐dex treatment (*P* = 0.022), prior number of regimens (*P* = 0.050), longer duration of Len‐dex treatment (*P* = 0.002), and achievement of ≥ VGPR response to treatment (*P* = 0.017). A multivariable analysis of the significant variables confirmed age at diagnosis (*P* = 0.005), duration of Len‐dex treatment (*P* = 0.026), and G‐CSF use (*P* = 0.029) as independent predictors of SPM.

### Hematological malignancies (MDS/AML)

Patients who developed MDS/AML had been previously exposed to oral cyclophosphamide or melphalan for a median of 7 months compared to 4.4 months in the non‐SPM group; however, this difference was not statistically different (*P* = 0.820). There was also no significant difference in number of prior treatment regimens, including ASCT. However, MDS/AML patients had less often received thalidomide (33% vs. 64%) and bortezomib (17% vs. 43%) than non‐MDS/AML patients, but due to small sample size neither comparison reached statistical significance, *P* = 0.20 and 0.24, respectively (Table 1). The percent of patients given concomitant granulocyte colony‐stimulating factor (G‐CSF) was higher in the MDS/AML group, (83% vs. 55%). Again, the difference was not statistically significant, likely due to the small sample size (*P* = 0.24). Previous radiotherapy had been administered to two out of six patients (33%) in MDS/AML group versus 25% in the non‐SPM group (*P* = 0.64).

All patients who developed AML/MDS had complex cytogenetics, except for one patient that had normal male karyotype 46XY. For this patient, no FISH cytogenetic analysis was available (Table 3). Abnormalities of chromosome 5, including deletion of the whole chromosome, were found in three out of six patients (50%), deletion of chromosome 18 was reported in three patients (50%), and 17p deletion in two patients (33%).

**Table 3:** Cytogenetics of multiple myeloma patients with secondary MDS/AML

Secondary diagnosis	ISS stage diagnosis	Time until relapse on Lenalidomide (months)	Cytogenetics	Deletions FISH	Additions (known)‐FISH	Additions (unknown)–FISH	Translocations– FISH
MDS/RAEB‐2	3	22.8	44~47, XY 46, XY[4]	−4, del (4) (q12), del(4) (q21)), −17, −18, −21	add (7) (q22), add (11) (q23), add (14) (q32)	+mar1, +mar2, +mar3, +mar4 [cp15]	
MDS/Plasma cell leukemia	2	30.1	45, XY 46, XY[2]	−13	–	+mar [cp2]	der (19) (p13.3;?) [16] der (19) r (19;?)
MDS	2	8.4	46 XY (normal)	Unknown	Unknown	Unknown	Unknown
MDS/SCC	Unknown	23.0	46, XX	−5, del (6) (p21.3[2]	add (17) (p11.2) add (17) (D5S721‐, D5S23−, EGR1+) (TP53−, D17Z1+)	+mar [6]	–
AML	1	64.0	43~45, XX	−5, del(7;17)(p10;q10) −14, −18	add (12)(p11.2),del(12)(p11.2p13) add (14)(p11.2)add (18)(q23), −20, i(21)(q10),add (22)(p11.2), add (22) (p13), +1~3r[cp12]	–	–
MDS	2	34.7	Unknown	5q−, −18	–	–	–

SPM, secondary primary malignancies; Dx, diagnosis; MDS, myelodysplastic syndrome; SCC, squamous cell carcinoma; ASCT, autologous stem cell transplant; FISH, fluorescent *in situ* hybridization; RAEB, refractory anemia with excess of blasts.

### Nonhematological malignancies

Comparing the NHM group versus the non‐SPM group, there was no significant difference in the number of prior treatment regimens, prior ASCT or the length of exposure to oral cyclophosphamide or melphalan before lenalidomide treatment (median of 4 months in both groups) (Table 1). All patients in the NHM group (100%) received G‐CSF as compared to 55% in the control group (*P* = 0.06). Previous radiotherapy had been given to three patients (60%) in NHM group versus 25% in the control group.

### Progression‐free and OS

**Figure 2:**
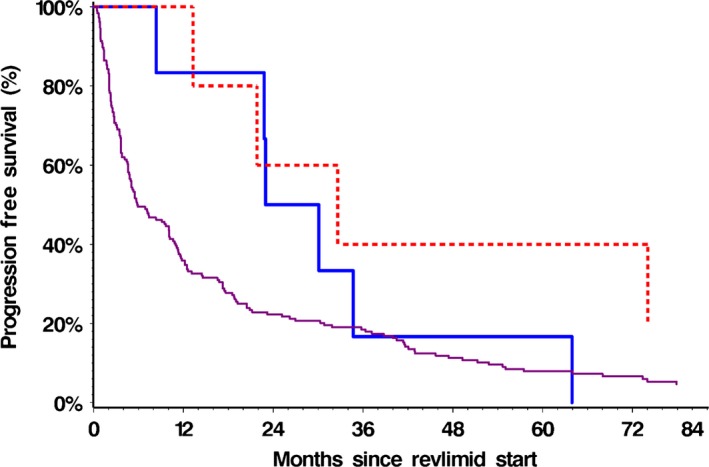
PFS from start of lenalidomide for MDS/AML, NHM and non‐SPM groups. Two‐year progression‐free survival was 50% (95% CI, 11.10–80.37%) in the MDS/AML group, 60% (95% CI, 12.57–88.18%) in the NHM group and 22.28% (95% CI, 16.58–28.53%) in the Non‐SPM group, Log‐rank *P* = 0.077 Wilcoxon *P* = 0.016.

The median PFS was significantly different between the SPM and non‐SPM groups, 30.1 months (95% CI 13.3–63.9) versus 5.9 months (95% CI 4.8–10.1), respectively (Log‐rank *P* = 0.029; Wilcoxon *P* = 0.004). The differences in PFS between MDS/AML, NHM, and non‐SPM groups were also statistically significant (Wilcoxon *P* = 0.004), Figure 2.

All six patients in the MDS/AML group died at a median of 7.4 months (range 0.89–13.19) from diagnosis of their SPM. One patient with RAEB‐2 progressed to AML, one died from progressive CHF requiring placement of a cardiac defibrillator without progression of MM or AML, one succumbed directly to AML progression, one with MDS progressed to plasma cell leukemia and one developed squamous cell carcinoma. The exact cause of death was unknown for one MDS patient, but there was no known progression of MM or AML. Two out of the five NHM patients were still alive at last follow‐up after completing 79 and 74 months of Len‐dex treatment. The patient diagnosed with urethral cancer had received 74 months of Len‐dex therapy before she was discontinued at the time of surgical resection and nephrectomy. The second patient, diagnosed with squamous cell carcinoma of head and neck, was still receiving Len‐dex (post‐79 cycles) at last follow‐up.

**Figure 3:**
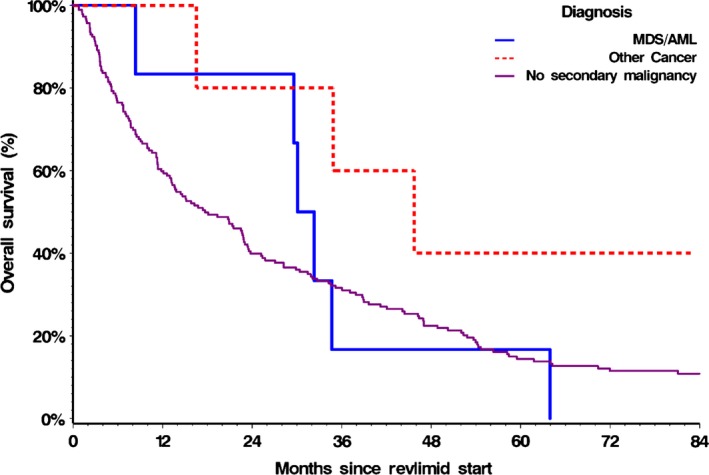
Overall survival (OS) from start of lenalidomide treatment for AML/MDS, NHM and non‐SPM groups. Two‐year OS was 83.33% (95% CI, 27.31–97.47%) in the MDS/AML group, 80% (CI 20.38–96.92%) in the NHM group and 39.89% (CI 32.74–46.93%) in the Non‐SPM group, Log‐rank *P* = 0.24 Wilcoxon *P* = 0.14.

The median OS from the start of lenalidomide therapy was 17.7 months (95% CI 13.2–22.9) for non‐SPM group, 31.2 months (95% CI 8.4–63.9) for MDS/AML group, and 45.7 months (95% CI 16.5 – no upper bound) for NHM group. There was no significant difference in OS between the three groups (*P* = 0.24). However, when the MDS/AML and NHM groups were combined as one SPM group and compared to the non‐SPM group, the OS survival was longer for the SPM group, log‐rank *P* = 0.17 and Wilcoxon *P* = 0.059 (Fig. 3).

## Discussion

As MM patients experience longer survival times, an increased risk of SPMs, including MDS/AML and solid tumors, has been observed. The etiology of this increased risk is multifactorial, but therapy‐related factors are contributory. There has been particular interest in the risk related to lenalidomide, one of the most effective anti‐myeloma agents available, prompted by the unanticipated increased incidence of SPM noted in several large trials utilizing single‐agent lenalidomide maintenance after induction therapy in elderly patients or after ASCT in younger patients 10, 11. Whether the use of lenalidomide with corticosteroids in the setting of RRMM is associated with an increased incidence of SPM has been a matter of debate.

The largest analysis examining this question pooled data from 11 clinical trials of lenalidomide‐based therapy involving 3846 patients with RRMM and reported an IR of 3.62 for all SPMs and an IR of 2.08 for invasive hematological and solid tumors 12. In a separate subset analysis of only the pivotal phase 3 trials of RRMM (*N* = 703), the overall IR of SPM was 3.98 with Len‐dex compared to 1.38 in placebo/dexamethasone. In a separate study, IR of SPM in a collective analysis of 313 patients that received the Len‐dex combination for more than 24 months was 2.35 14, 15. Our results showed an IR of 2.37 and a cumulative IR of 5.24% for all SPMs diagnosed while on treatment with Len‐dex. Further, our findings of an IR for SPM of 0.86 per 100 patient‐years from MM diagnosis in patients on the Len‐dex regimen are consistent with the age‐adjusted incidence rates of 0.8 for invasive cancers among persons 55–60 years of age, as reported in the “Surveillance, Epidemiology and End Results Program” (SEER program) 16.

Not surprisingly, patients who developed AML/MDS had a longer median duration of previous exposure to the oral alkylating agents, cyclophosphamide and melphalan—agents known to carry leukemogenic potential. Recent meta‐analysis showed increased risk of SPM in patients who underwent ASCT 17. Even though majority of our patients had previous ASCT, we did not see any association between previous single or tandem ASCT and increased risk of SPM. However, possibly due to the small patient numbers, this parameter did not reach statistical significance. Further analysis of larger cohorts of patients may address this question. Other factors including advanced age and light chain isotype of MM may predispose to SPM. However, due to small patient numbers in each group, we could not make a conclusion. Neutropenia is a common side effect of lenalidomide. Our study demonstrated that the concurrent use of G‐CSF was associated with a significantly higher risk for all SPM with a hazard ratio of 7.82. The use of G‐CSF in our study was more liberal than in usual practice, as our policy at PMH at the time was to try to maintain a lenalidomide dose of 25 mg throughout relapse using growth factor support rather than to reduce the dose if neutropenia occurred 18, 19. Higher usage of G‐CSF has also been associated previously with an increased risk for SPMs, including myeloid malignancies, in other cancers treated with conventional chemotherapy without lenalidomide 20. On the other hand lenalidomide may induce a higher degree of neutropenia in MM patients who have either underlying, or not yet diagnosed MDS. In this case, lenalidomide may “stress” vulnerable bone marrow and result in increased need of G‐CSF. Such patients who develop neutropenia while on lenalidomide and require G‐CSF may be at higher risk for MDS and should be assessed earlier for this entity. More recently, we have modified our approach to lenalidomide‐related neutropenia, and now favor dose reduction rather than the routine—and often prolonged— use of G‐CSF to maintain a dose of 25 mg. Even though the absolute overall risk appeared to be small, the relationship between G‐CSF use and SPM should nevertheless be considered in clinical decisions with regard to the use of growth factors.

In our study five out of the six patients with MDS/AML had complex cytogenetic abnormalities. In a recent report of MM patients who developed therapy‐related MDS or AML, complex cytogenetic abnormalities were reported in 79% of MDS and 82% of AML patients 21. Interestingly, these five patients presented with a monosomal phenotype including deletions of chromosomes 5, 14, 18, 13, and 21. A monosomal karyotype has a negative prognostic impact in AML patients with a complex karyotype, and in AML with MDS‐related cytogenetic abnormalities 20.

Until recently, the survival of MM patients was relatively short, a feature which may have contributed to lower reported incidence of SPMs. The introduction of novel agents such as lenalidomide has significantly improved OS of MM patients. With RRMM patients living longer, it is reasonable to expect a rise in IR of SPM, because of increasing patient age, factors intrinsic to MM and higher exposure to multiple lines of therapy. In our study, patients who developed SPM had the best response rates, PFS, and OS. This may be either from a prolonged exposure needed to develop SPM or longer exposure to lenalidomide. These observations highlight the benefits of lenalidomide in the management of RRMM, and likely explain to a large extent why OS was not affected by development of MDS/AML or NHM in our study.

We found that the median time to MDS/AML development was 6.7 years post diagnosis of MM. This interval is consistent with the recently published report from MD Anderson Cancer Program in which the median time from MM diagnosis to therapy‐related myeloid neoplasms, including MDS/AML, was 7 years 20. On the other hand, we observed the development of AML/MDS relatively early after the initiation of Len‐dex, with a median of only 25 months. Other reports have noted that MDS was not seen in patients experiencing long‐term benefit with Len‐dex therapy,that is, those with a PFS greater than 3 years. One could speculate that development of SPM occurs relatively early in the course of Len‐dex therapy because the full protective immunomodulation effect or deepening responses from use of lenalidomide treatment are achieved with continued treatment and are not fully realized early on 22, 23.

The finding that the overall IR of SPMs from diagnosis observed in our patients with RRMM receiving Len‐dex was comparable with the control population in the SEER data is reassuring. However, when assessed from the initiation of Len‐dex, SPMs do develop with an IR of 2.37 per 100 patient‐years and a cumulative incidence rate of 5.24%, and efforts to minimize these complications are desirable. Longer previous exposure to oral alkylators and longer duration of lenalidomide therapy were more common in patients who developed MDS/AML. Adverse risk factors predisposing to SPM in multivariate analysis include advanced age and longer duration of lenalidomide therapy. Patients with these features should have a low threshold for evaluating new signs and symptoms that could reflect the development of SPM. Whether or not the link between G‐CSF use and higher risk of MDS that we observed reflects cause or effect cannot be ascertained from our study. However, an increased awareness of the potential for the occurrence of SPMs has led to more rigorous prospective monitoring in recent myeloma clinical trials, and further information about the risk will be forthcoming as these studies mature.

## Conflict of Interest

D.R. Honoraria and Research Funding from Otsuka, BMS, Merck, Novartis, Millennium, Celgene, Janssen, Onyx; S.T. received honoraria from Celgene, Amgen, Novartis, BMS, Stock ownership of Celgene, and on the Speaker bureau of Celgene, Amgen; R. T. received honoraria from Celgene, Janssen Ortho, Amgen; V.K. received honoraria from Celgene, Amgen, Lundbeck, Roche, and Janssen Ortho; C.C. received prior honoraria, research funding, and consultancy fee from Celgene. The remaining authors declare no competing financial interests.
